# Multi-Tracking Sensor Architectures for Reconstructing Autonomous Vehicle Crashes: An Exploratory Study

**DOI:** 10.3390/s24134194

**Published:** 2024-06-27

**Authors:** Mohammad Mahfuzul Haque, Akbar Ghobakhlou, Ajit Narayanan

**Affiliations:** School of Engineering, Computer and Mathematical Sciences, Auckland University of Technology, Auckland 1042, New Zealand; akbar.ghobakhlou@aut.ac.nz (A.G.); ajit.narayanan@aut.ac.nz (A.N.)

**Keywords:** tracking architecture, GOSPA, sensor fusion, multi-sensor, performance evaluation, SMTPE, autonomous vehicle, crash reconstruction

## Abstract

With the continuous development of new sensor features and tracking algorithms for object tracking, researchers have opportunities to experiment using different combinations. However, there is no standard or agreed method for selecting an appropriate architecture for autonomous vehicle (AV) crash reconstruction using multi-sensor-based sensor fusion. This study proposes a novel simulation method for tracking performance evaluation (SMTPE) to solve this problem. The SMTPE helps select the best tracking architecture for AV crash reconstruction. This study reveals that a radar-camera-based centralized tracking architecture of multi-sensor fusion performed the best among three different architectures tested with varying sensor setups, sampling rates, and vehicle crash scenarios. We provide a brief guideline for the best practices in selecting appropriate sensor fusion and tracking architecture arrangements, which can be helpful for future vehicle crash reconstruction and other AV improvement research.

## 1. Introduction

The adoption of autonomous vehicles (AVs) is increasingly based on Advanced Driver Assistance Systems (ADASs) facilities, with people enjoying the comfort and artificial intelligence-based services while driving autonomous vehicles. Autonomous vehicle development has reached L3 (level 3), where a driver can ‘eyes off’ in a highly automated driving environment. This ongoing development is now at level 4 and rapidly heading toward level 5. At level 4, a driver can focus on other tasks rather than driving; in the future, at level 5, everything can be controlled by the fully automated driving environment [1,2]. Though the developments of ADASs are significant, there are accident cases reported where AVs sometimes cannot avoid collisions. There could be different reasons for those crashes. Researchers continue to examine the possible contributing factors behind AV crashes by analyzing the existing crash datasets [3,4]. Some contributing factors to AV crashes include inaccurate perception of road obstacles and adverse environmental conditions such as rain and fog [5]. In light of this, the development of perception mechanisms for AVs is continuous. Perception is one of the fifth components of autonomous vehicle navigation and autopiloting. The other navigation components are localization, planning, decision-making, and dynamic vehicle control [6,7]. For an AV, perception performs sensing like a human driver. In the same way, sensors are the primary inputs that scan the vehicle’s surroundings for objects. A sensor’s perception has limitations, which can be mitigated by fusing multiple sensors [8]. Moreover, combining radio detection and ranging (radar), camera, or light detection and ranging (LIDAR) for sensor fusion can enhance the detection performance for autopiloting by adding efficiency and reliability [9]. Sensor-based object detections are processed in a tracker. A tracker produces motion states to monitor other vehicles’ movements, such as position, velocity, and orientation. Researchers continuously develop new trackers and improve tracking algorithms to advance AV developments. For example, the authors improved the target tracking performance by introducing the optimal geometry and motion coordination for multi-sensor target tracking [10]. In [11], a lane detection algorithm is developed using multi-sensor fusion to improve lane boundary tracking in curvature. In addition, algorithms developed for better tracking of crowded scenes [12] solved the lag smoothing problem to track a turning target [13] and enhanced the path tracking of a vehicle [14]. Similarly, MATLAB and Simulink provide options for AV researchers to examine their tracking algorithms using two types of architectures. Central-level tracking is the first type of architecture, and the second type is track-to-track fusion. A central-level tracking architecture performs better than the other type because it receives object detection information directly from vehicle sensors. In contrast, the second or distributed type of tracking architecture receives sensor-level tracks as the input to its track-to-track fuser (a Simulink block that takes tracks as input from object trackers, not sensor data directly) for track-level fusion. A track contains target tracking information for a specific time step, whereas target tracking from a complete trajectory contains multiple tracks. Tracks produced from a tracker have lower data sizes than unprocessed sensor data. Therefore, distributed architecture performs better in resource capability limitations, such as transmission bandwidth, computation power, and the execution of multiple tracking algorithms [15,16].

The availability of multiple types of sensors for sensor fusion, object trackers with different algorithms, and tracking architectures is now evident. As a result, researchers may develop AVs by arranging the tracking architecture in various ways. However, there is no standard research method or benchmarks for experimenting with different architectures apart from an arbitrary selection of sensor fusion-based tracking configurations. This is particularly important when needing to reconstruct an AV crash for forensic and investigative purposes, where there is a need for assurance that all the important information has been captured by the sensors and that no important information has been lost. The main problem is the relative lack of published research papers in AV crash reconstruction using multi-sensor-based sensor fusion in comparison to papers dealing with sensors and their architectures. Therefore, developing a simulation method for evaluating different architectures for AV crash reconstruction is essential. To this end, this exploratory study asked the following research question (RQ):

RQ: What simulation method can be developed to select architectures using multi-sensor fusion for AV crash reconstruction?

This research aimed to develop a novel simulation method to decide on the best tracking architecture for AV crash reconstruction. This study examined all types of crash scenarios (head-on crash, rear-end crash, and side-impact crash) using the Crash Injury Research Network (CIREN) dataset [17]. The primary purpose of the simulated experiment for this study was to evaluate the tracking performance in different crash scenarios. Additionally, the sensor fusion-based tracking performance results can be used as the input for AV crash reconstruction-related research. The rest of this paper is arranged in the following sections. Section 2 briefly reviews the literature to identify the ongoing developments and gaps. The proposed simulation model is presented in Section 3. Section 4 describes the results. Section 5 discusses the tracking performance evaluation. Finally, a conclusion of this research is presented in Section 6.

## 2. Related Works

Xiaobin et al. proposed a combined optimal sub-pattern assignment (C-OSPA) metric to evaluate the performance of multi-target tracking (MTT) [18]. The authors found several improvement initiatives to overcome the optimal sub-pattern assignment (OSPA) limitations, such as detecting labeling errors and adding tracking quality for accuracy measurement. They solved the angular error issues in C-OSPA to obtain better state estimation results for supplementing the existing OSPA improvements. Their proof of a theoretical concept of performance metrics can evaluate MTT. However, it was not comprehensive, as the authors performed mathematical simulations and did not present object detections or did not perform any trajectory evaluation using a real scenario.

A tracking performance evaluation method was proposed to detect better center locations of objects [19]. The authors used image pixels to correct an object’s center location with the help of an adaptive threshold and background suppression. This image processing-based approach used data from the camera sensor to label object detections using an annotation box and bounding box with an improved center location. Their approach can track vehicles using a single sensor for detection, which is its limitation.

The authors conducted a performance evaluation study on an intelligent trajectory tracking controller applying three algorithms [20]. The evaluation variables were tracking performance, algorithm execution time, and steering errors. The authors considered lateral position and heading angle errors as steering errors. In their study, they did not present the perception mechanism of the trajectory environment, hence raising repeatability issues in relation to their solution.

Yoonsuk et al. developed a model predictive control-based algorithm utilizing sampling time to take inputs such as lateral velocity steering angle to improve path-tracking performance [14]. The authors experimented with their model using four different sampling times in a MATLAB-based simulation experiment. Their proposed algorithm performed better in smaller sampling times. They tested their algorithm using a vehicle traveling scenario to track the kinematics of the vehicle itself. In real scenarios, other vehicles may exist on the road, which must be detected for safe path tracking if the solution targets AV development. Therefore, their path-tracking algorithm needs further improvements.

Rahmathullah et al. solved the issues of the optimal sub-pattern assignment (OSPA) metric and named it Generalized OSPA (GOSPA) to achieve optimization over assignments [21]. The GOSPA detects estimated, missed, and false targets by penalizing localization errors. In addition, it supports a random fine set (RFS) of targets for evaluating the MTT performance. The authors further extended the GOSPA metric by measuring the gap between two sets of trajectories via penalized trajectory switching [22].

Another recent initiative to improve the GOSPA metric was presented in [23]. The authors proposed an evaluation method for multi-target tracking. This method included temporal dimension specifics to evaluate the anticipated trajectories by comparing them with actual trajectories. It can evaluate tracking accuracy, continuity, and clarity based on errors such as localization, missed, switching, and false trajectory errors. Therefore, these features enable a new way to select comparatively better MTT algorithms for higher tracking performance.

In an overview study, the authors revisited visual tracking measures and evaluated the performance measures for tracking performance. The authors evaluated 16 trackers using 25 video scenarios to present the performance results accurately and robustly. Minimizing the number of performance measures from a list of enormous measurement variables for tracking evaluation was the main idea behind visual tracking evaluation reliability [24].

A vehicle tracking algorithm was developed based on the extended Kalman filter for tracking the trajectories of a platoon of vehicles in [25]. The tracking performance of a vehicle platoon was evaluated using external numerical analysis, applying improved position estimation through an array of antennae and a transmitting infrastructure built into the road system. This vehicle-to-infrastructure-dependent tracking method requires data produced from external sources rather than relying on internal AV data alone.

Alai and Rajamani developed a sensor fusion-based, cost-effective vehicle tracking system that can detect obstacles in real time to avoid possible crashes [26]. Their algorithm improved the target vehicle’s corner detection through multiple sensor fusion. However, this solution is limited to micromobility vehicles such as e-scooters and deals only with front-end target tracking.

Many researchers utilized tracking performance thresholds to improve the results of their proposed solutions for vehicular developments. Such initiatives included vehicular platoon management [27] and controlling the tracking performance of mobile robots [28].

As can be seen from the literature review, several contributions have been made to developing new tracking performance evaluation and enhancement metrics. Researchers have already developed algorithms and evaluation metrics for deciding on an appropriate simulation setup for performance tracking evaluation. Most importantly, a need for a standard method to select an appropriate experimental setup that can produce better results still exists. To this end, this research proposes a repeatable simulation method to decide on the best tracking architecture for AV crash reconstruction.

A summary of the related works is presented in Table 1.

## 3. The Proposed Method and System Design

**Figure 1:**
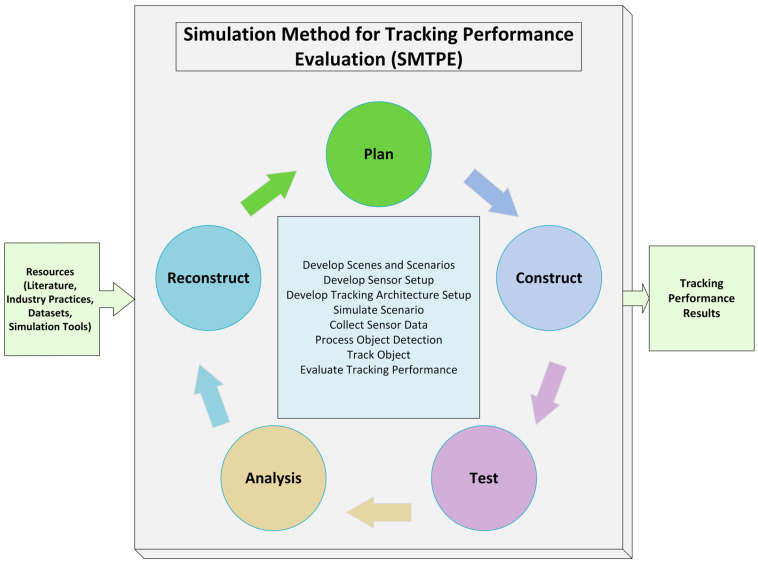
Proposed simulation method for tracking performance evaluation (SMTPE): a repeatable solution for object tracking performance evaluation in AV development.

The perception of the surrounding environment is the most critical factor for AV technology. In order to achieve a repeatable solution for tracking performance evaluation, this research proposed a simulation method for tracking performance evaluation (SMTPE) to select the appropriate sensor fusion and tracking architecture arrangements, as shown in Figure 1. Primarily, this research used SMTPE to obtain the best experimental setup for vehicle crash reconstruction research.

The primary process of SMTPE is iterative and it will continue until a decision can be made on the evaluated performance of the object tracking. Coupled with this, planning, constructing, testing, analyzing, and reconstructing are the main elements of SMTPE. The plan is the first element of the iterative method for creating a simulation design, which requires the analysis of resources, such as the simulation tools and datasets. The construction element helps develop trajectory scenarios, tracking architecture, and sensor setup according to the design using selected simulators. The main tasks of the test element are executing simulations and fixing errors. The simulated results are then analyzed for performance evaluation. The reconstruction is the last element of the iterative cycle to confirm the developed simulation’s repeatability and helps to understand the AV crash scenario thoroughly. If an optimal solution is achieved using the iterative method, the SMTPE process will stop execution; otherwise, it will continue until a satisfactory result is achieved. Using these elements, SMTPE performs the core processing tasks: develop the scenes and scenarios, develop the sensor setup, develop the tracking architecture setup, simulate the scenario, collect the sensor data, process the object detection, track objects, and evaluate the tracking performance. Finally, the output section of SMTPE is responsible for delivering a provision for deciding on the best tracking setup for further experiments. The following Section 3.1 describes the SMTPE process in detail.

### 3.1. Process Flow of SMTPE

**Figure 2:**
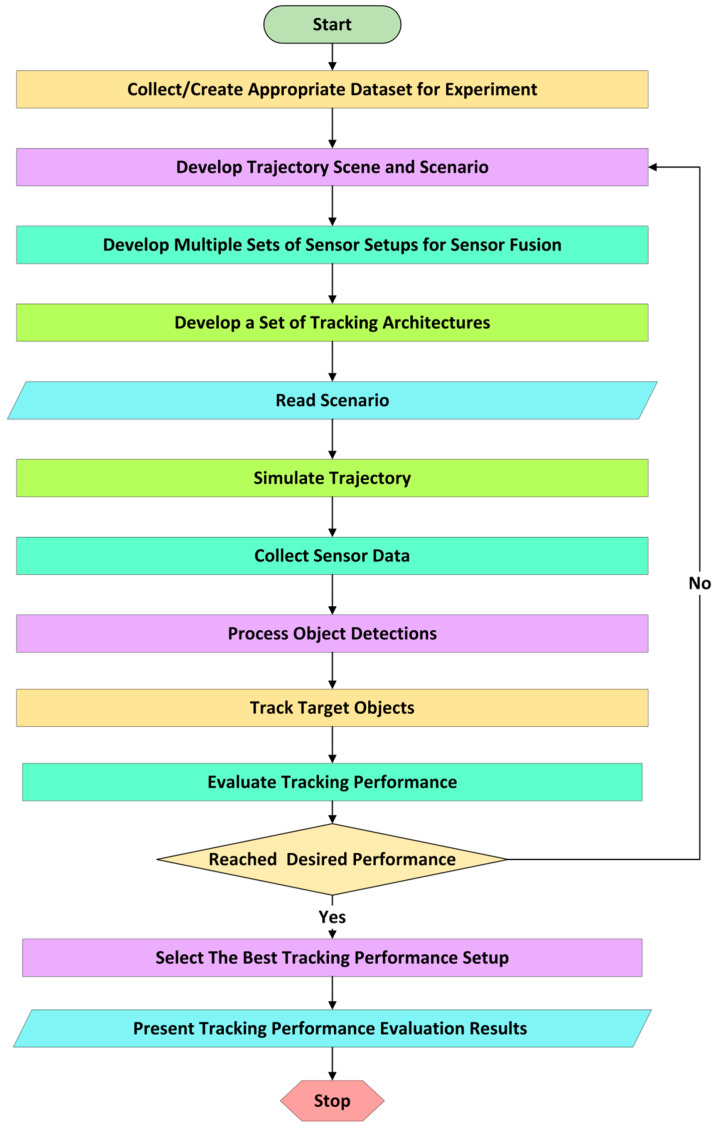
The process flow of SMTPE: selecting the best tracking performance setup for AV crash reconstruction development.

A process flow for SMTPE has been illustrated in Figure 2. The process starts by collecting the appropriate datasets or recording a dataset from a real scenario. After analyzing the collected trajectory data, the next step is developing a plan for creating the required scenes or scenarios. In this step, the ego vehicle (the vehicle producing and storing the data), other vehicles, roads, and other objects (such as pedestrians and bicyclists if needed) are constructed similarly to the acquired dataset. A trajectory scenario consists of many objects, and the properties of these objects must be assigned accurately to achieve better experimental results.

The present AV technology uses sensors to obtain the surrounding perceptions for autopiloting. Precise knowledge is needed to deal with sensing the surroundings in the different environments of the trajectories. Indeed, expertise on the advantages and limitations of sensors is also essential to dealing with real-world environments such as perception at night, rain, fog, or driving on a mountainous road. Similarly, experimenting with different sensors (such as radar, camera, and LIDAR) with multiple orientation-based setups can help select the best experimental setup for planned research. Concurring with the above statements, SMTPE’s step of developing multiple sets of sensor setups generates data to feed in for the next step.

The object tracking architecture (TA) is a recently introduced feature for AV development. The TA helps to experiment with different trackers (a tracker tracks target objects by following their movements) and algorithms that produce target tracking. Indeed, the core processing is performed in the sensor fusion subsystem by the TA step to compare the outputs of different trackers and the performance of tracking algorithms. Therefore, arranging a set of TAs for experiments helps select a better TA than experimenting with a single TA.

A trajectory scenario reading is a prerequisite for a simulator. Therefore, the scenario reading and simulation steps must be executed one after another to collect simulated data from the reconstructed scenario.

The core function of a sensor is sensing and producing object detections. Object detections need further processing to feed into a tracker, for example, aggregating object detections from sensors and adding localization information. In SMTPE, the object detection step performs this processing.

Selecting the appropriate tracking algorithm and object tracker requires insight into the tracking algorithm and tracker functionalities. Experimenting with different arrangements of tracking algorithms using trackers can give the option to select a better TA. The track-target-object step of SMTPE provides this option. In addition, the simulation performance evaluation of object tracking needs to be iterative to reach a desired performance so that there is confidence that enough information has been obtained from the scenario and trajectory scenes for setup evaluation to proceed. A tracking metric serves this performance evaluation and must be well understood and researched to perform this desired performance function. For example, the GOSPA metric is used in many studies, and researchers continuously enhance its features [21,22,23].

The best tracking setup is discovered through a performance threshold value to evaluate individual experimental setups first. For our experiments, we set a 90% target tracking accuracy. Then, ranking all the experiments’ setups was performed to obtain the best one. Finally, if the evaluation cycle delivers the desired performance, the output of SMTPE’s evaluation and ranking steps is presented to a researcher. Otherwise, the best architecture searching process redirects to the scenario development step of SMTPE. This iterative process continues until an optimal result is achieved.

### 3.2. Formulas for Developing SMTPE

The following subsections deal with the mathematical representations of tracking performance evaluation.

#### 3.2.1. Tracking Objects

The Kalman filter uses a chain of detections or measurements from the vehicle sensors to estimate an object’s state based on its motion model. An object’s state contains position, velocity, acceleration, or turn rate in a motion model. An extended Kalman filter is a nonlinear version of the Kalman filter that linearizes the state equation and measurement equation. Linearization helps create the linear formatted state and state covariance, which needs Jacobians of the state and measurement equations [29].

In this study, the extended Kalman filter was initialized with a constant velocity motion model and a constant velocity measurement model for the sensor fusion-based object tracking. An object tracker, such as a joint probabilistic data association tracker, can utilize a filtering function to produce estimated states as vector values and state estimation error covariance as a matrix to track objects. As a filtering function, the extended Kalman filter uses the state transition function (a function returns the state vector at a time step using the state vector of the previous time step), measurement function (a function returns the output measurement for each time step), and initial states as input parameters in a tracker based on the data fused from the sensors [29]. These input values are then processed for each time step by an extended Kalman filter algorithm to serve the state estimation of a target object. The three-dimensional (3-D) constant velocity model’s state equation [30] is shown in (1). Here, $x_{k},\;y_{k},$ and $z_{k}$ are the position coordinates of vehicle *k*, $v_{x,k},v_{y,k},$ and $v_{z,k}$ are corresponding velocities, and T is the time step of the state transition.

(1) $$\left[\begin{array}{c} x_{k+1}\\ v_{x,k+1}\\ y_{k+1}\\ v_{y,k+1}\\ z_{k+1}\\ v_{z,k+1} \end{array}\right]=\left[\begin{array}{cccccc} 1 & T & 0 & 0 & 0 & 0\\ 0 & 1 & 0 & 0 & 0 & 0\\ 0 & 0 & 1 & T & 0 & 0\\ 0 & 0 & 0 & 1 & 0 & 0\\ 0 & 0 & 0 & 0 & 1 & T\\ 0 & 0 & 0 & 0 & 0 & 1 \end{array}\right]\left[\begin{array}{c} x_{k}\\ vx_{k}\\ y_{k}\\ vy_{k}\\ z_{k}\\ vz_{k} \end{array}\right]$$

The simplified 3-D state vector structure is shown in (2).

(2) $$\mathrm{State}\;=\left[x;\;vx;\;y;\;vy;\;z;\;vz\right]$$

#### 3.2.2. State Update Model

The extended Kalman filter maintains prediction and correction in a loop that uses the nonlinear state update and measurement functions in a time series cyclic order [29]. A predicted state can be computed as a function of a previous state $x_{k}$, controls $u_{k}$, noise $w_{k}$, and time t, as shown in (3).

(3) $$x_{k+1}=f\left(x_{k},u_{k},w_{k},t\right)$$

The partial derivatives of the Jacobian with respect to the previous state can be expressed in (4).

(4) $$F^{\left(x\right)}=\frac{\partial f}{\partial x}$$

Function (5) presents the Jacobian of the predicted state concerning the noise.

(5) $$F^{\left(w\right)}=\frac{\partial f}{\partial w}$$

Functions (3)–(5) are more straightforward when the noise is additive in the state update equation as shown in (6). Here, $F^{\left(w\right)}$ is an identity matrix. The previous state $x_{k}$, controls $u_{k}$, and noise $w_{k}$, are used to compute the predicted state $x_{k+1}$ at time t.

(6) $$x_{k+1}=f\left(x_{k},u_{k},t\right)+w_{k}$$

#### 3.2.3. Measurement Model

A measure can be calculated using the nonlinear function of the state and the measurement noise, as shown in Equation (7).

(7) $$z_{k}=h\left(x_{k},v_{k},t\right)$$

The Jacobian of the measurement concerning the state is presented in Equation (8).

(8) $$H^{\left(x\right)}=\frac{\partial h}{\partial x}$$

The Jacobian of the measurement concerning the measurement noise is shown in Equation (9).

(9) $$H^{\left(v\right)}=\frac{\partial h}{\partial v}$$

Functions (7)–(9) are more straightforward when the noise is additive in the measurement equation as shown in (10). Here, $H^{\left(v\right)}$ is an identity matrix. The measurement $z_{k}$ of the vehicle *k* is calculated using control $x_{k}$ and measurement noise $v_{k}$ at time t.

(10) $$z_{k}=h\left(x_{k},t\right)+v_{k}$$

#### 3.2.4. Generalized Optimal Sub-Pattern Assignment Metric

The generalized optimal sub-pattern assignment (GOSPA) metric is a Simulink block introduced in MATLAB version R2021a. This GOSPA metric evaluates the tracking algorithm’s performance, accepting tracks generated from the object detections and known truths as input parameters. In addition to the GOSPA score, it can present switching errors, localization errors, missed target errors, and false track errors. The algorithms of the GOSPA metric calculation [31] are provided below.

Equation (11) contains a set of truths at time $t_{k},$ whereas Equation (12) represents a list of tracks at time $t_{k}$. The GOSPA metric needs three input parameters to evaluate the tracking performance. These are confirmed tracks produced by a tracker, converted coordinates (positions) of the target vehicle (from the vehicle’s coordinates to world coordinates), and ego (the vehicle performs the perception using sensors) vehicle’s positions (world coordinates). The truth data, such as the coordinates of the target vehicle and ego vehicle, are extracted from the trajectory scenario by the vehicles, whereas a tracker produces the list of tracks in the sensor fusion system. So, the truth date of Equation (11) and the list of tracks of Equation (12) are provided by the above methods. These tracks and truths are used as the input to calculate the GOSPA as shown in Equation (13).

(11) $$X=\left(x_{1},\;x_{2},\ldots ,x_{m}\right)$$

(12) $$Y=\left(y_{1},\;y_{2},\ldots ,y_{n}\right)$$

(13) GOSPA=[∑i=1mdcpxi,yπi+cpαn−m]1p

Here, *m* ≤ *n*, *d_c_* is the cutoff-based distance, $y_{\pi \left(i\right)}$ represents the track assigned to truth *x_i_*, and *α* denotes the error due to cardinality mismatch. The cutoff-based distance *d_c_* is defined in Equation (14) as:

(14) $$d_{c}\left(x,y\right)=\min\left\{d_{b}\left(x,y\right),c\right\}$$

Here, *c* is the cutoff distance threshold, *p* is the order of the metric, and $d_{b}\left(x,y\right)$ is the base distance between the track and truth. When *α* = 2, the GOSPA metric can simplified as shown in Equation (15):

(15) GOSPA=[locp+missp+falsep]1p

Here, localization error ($loc^{p}$), missed targets ($miss^{p}$), and false targets ($false^{p}$) are calculated using Equations (16)–(18).

(16) loc=[∑i=1hdbpxi,yπi]1p

(17) miss=c21p(nmiss)1p

(18) false=c21p(nfalse)1p

Here, *h* is the number of nontrivial assignments, and $n_{miss}$ and $n_{false}$ are the numbers of missed targets and false tracks, *p* is the order of the metric, *c* is the cutoff distance, $d_{b}$ is the base distance between the track and truth, $y_{\pi \left(i\right)}$, and represents the track assigned to truth x_i_.

### 3.3. Crash Dataset for Tracking the Performance Evaluation and Classification of the Crash Type

The causes of collisions between AVs and conventional vehicles have been presented in a study where vehicle crashes were categorized as rear-end crashes, sideswipe crashes, and other crashes (head-on or hitting an object) [23,32]. The Crash Injury Research Engineering Network (CIREN) reported the planes of impacts or vehicle damage from crashes as front, back (rear), left, right, top, and undercarriage [17]. In this research, crashes between two vehicles were classified into four types. These are as follows:

1. Front crash (the ego vehicle and the other vehicle are traveling in the same direction)
2. Head-on crash (the ego vehicle and the other vehicle are traveling in opposite directions)
3. Rear-end crash (the ego vehicle and the other vehicle are traveling in the same direction)
4. Side-impact crash (the other vehicle can hit from any direction the ego vehicle’s left or right side).

**Figure 3:**
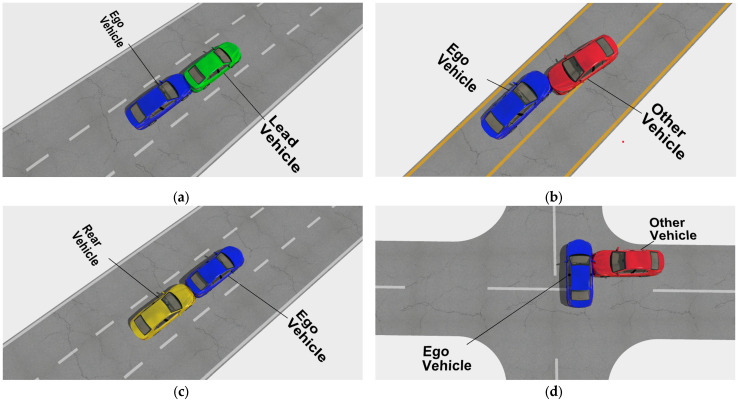
Classification of vehicle crashes: (**a**) front crash; (**b**) head-on crash; (**c**) rear-end crash; (**d**) side-impact crash.

Pre-crash (cut-in, conflict, potential crash) and crash events can be observed from the vehicle trajectory for each type of the above vehicle crashes. Figure 3 shows the front (Figure 3a), head-on (Figure 3b), rear-end (Figure 3c), and side-impact (Figure 3d) crashes.

### 3.4. Experimental Setup for Multi-Sensor-Based Surround Vehicle Sensor Fusion

This research developed the proof of concept of the proposed SMTPE using MATLAB (R2023b), Simulink (R2023b), and their tools (Driving Scenario Designer app, Navigation toolbox, and Automated Driving Toolbox). The experiment was developed using a machine with Windows 11 Pro (64-bit operating system, version 23H2), 11th Gen Intel Core i7 processor, and 16 GB RAM. The primary purpose of the simulated experiment for this study was to evaluate the tracking performance in different crash scenarios. The MATLAB’s Driving Scenario Designer app reconstructs the crash scenarios using the CIREN dataset [17]. All the sensor arrangements are first tested using this app and then reconstructed using the Simulink software. Three types of crash scenarios were reconstructed from the CIREN dataset. These were:

- Head-on crash (CIREN accident no 664 or CIREN-664);
- Rear-end crash (CIREN accident no 816 or CIREN-816);
- Side-impact crash (CIREN accident no 226 or CIREN-226).

The experimental setup subsection was split into sensor setup and tracking architecture setup.

#### 3.4.1. Sensor Setup

Usually, AV technology uses vehicle sensors to sense the AV’s surroundings. Radar, cameras, and LIDAR are the most popular sensors that scan the vehicle’s surroundings for perception. Besides the sensing capability, every sensor has some limitations. These limitations can be improved by fusing multiple sensors. Moreover, combining radar, cameras, or LIDAR for sensor fusion can enhance AV driving. This study utilized different arrangements of radar, cameras, and LIDAR to create two setups of multi-sensor-based surround vehicle sensor fusion. These multi-sensor-based surround vehicle sensor fusion setups were as follows:

Sensor Setup 1 (S1): The sensor setup 1 or S1 consisted of three radar sensors, five cameras, and one LIDAR sensor. The camera sensing fully covered the ego vehicle’s surroundings, which was achieved by positioning five cameras in the ego vehicle. Three radars were positioned: one at the front, one at the front left side of the vehicle, and another at the front right side of the vehicle. The LIDAR was placed at the center of the ego vehicle. These sensor setups are shown in Table 2.

**Figure 4:**
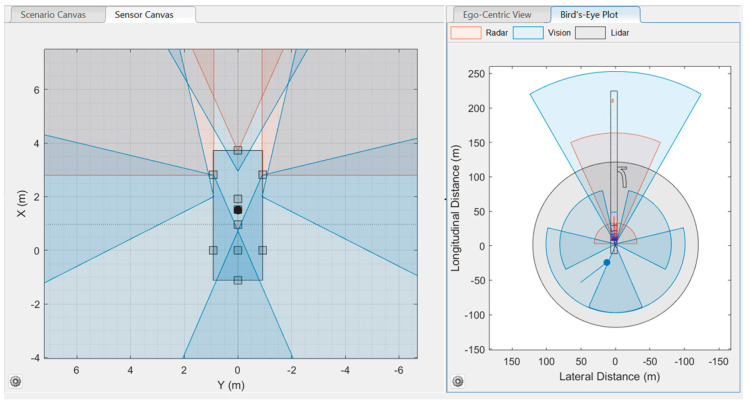
Sensor setup 1 (S1): three radars, five cameras, and one LIDAR.

The Driving Scenario Designer’s view of the developed S1 is shown in Figure 4.

Sensor Setup 2 (S2): The sensor setup 2 or S2 contained six radar sensors, two cameras, and one LIDAR sensor. The radars spanned the ego vehicle’s full surroundings for the S2. The ego vehicle had one camera at the front and another at the rear, mounted to fuse with the other sensors. The LIDAR was placed at the center of the ego vehicle. These sensor setups are shown in Table 3.

**Figure 5:**
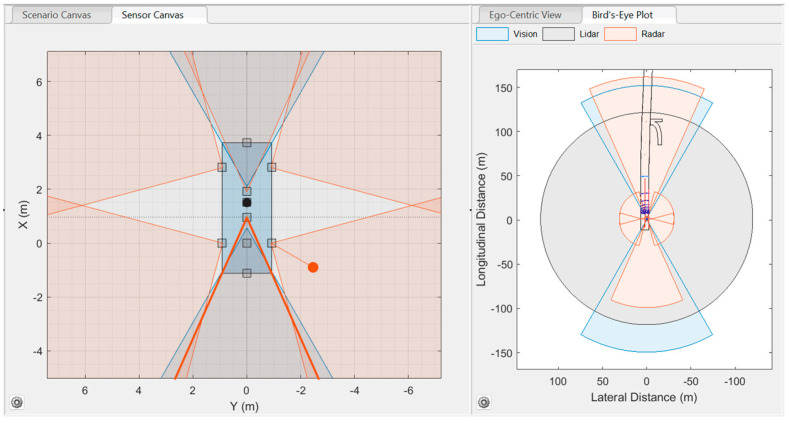
Sensor setup 2 (S2): five radars, two cameras, and one LIDAR.

The Driving Scenario Designer’s view of the developed S2 is shown in Figure 5.

#### 3.4.2. Tracking Architecture (TA) Setup

**Figure 6:**
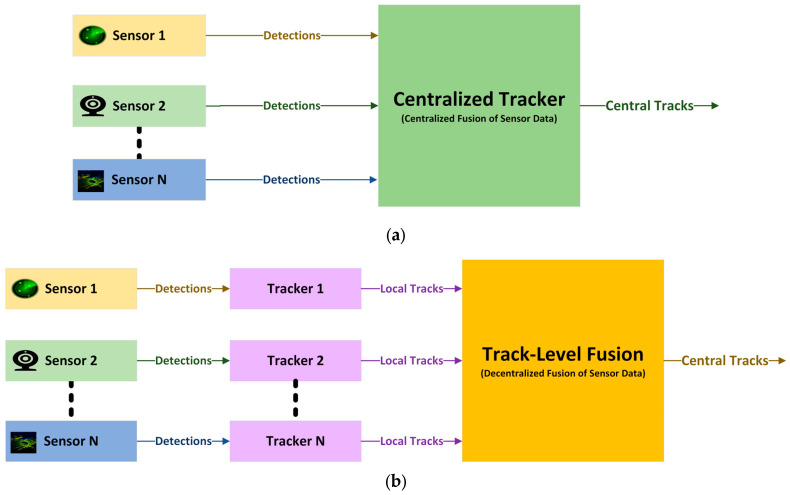
Tracking architectures: (**a**) centralized tracking architecture; (**b**) decentralized tracking architecture.

The multi-object tracker tracks the target vehicles to deliver the confirmed tracks. It produces confirmed tracks from a tracker that include estimated states and state covariances of the target vehicles. The estimated states contain the target vehicles’ kinematic information, such as position, velocity, and orientation, whereas the state covariances represent the uncertainty in the filtered states. The multi-object tracker uses a filtering algorithm to create the confirmed tracks. A central-level tracking architecture can perform better than a track-to-track fusion architecture because a central-level tracker receives object detection information directly from the vehicle sensors. The decentralized tracking architecture receives sensor-level tracking as the input to its track-to-track fuser for track-level fusion. The tracking architectures are presented in Figure 6. The centralized tracking architecture is presented in Figure 6a, and the track-to-track fusion or decentralized tracking architecture is presented in Figure 6b. Applying these two architectures, we constructed three Tas (two centralized, one decentralized) as illustrated in Table 4, differing in terms of the sensors used.

**Figure 7:**
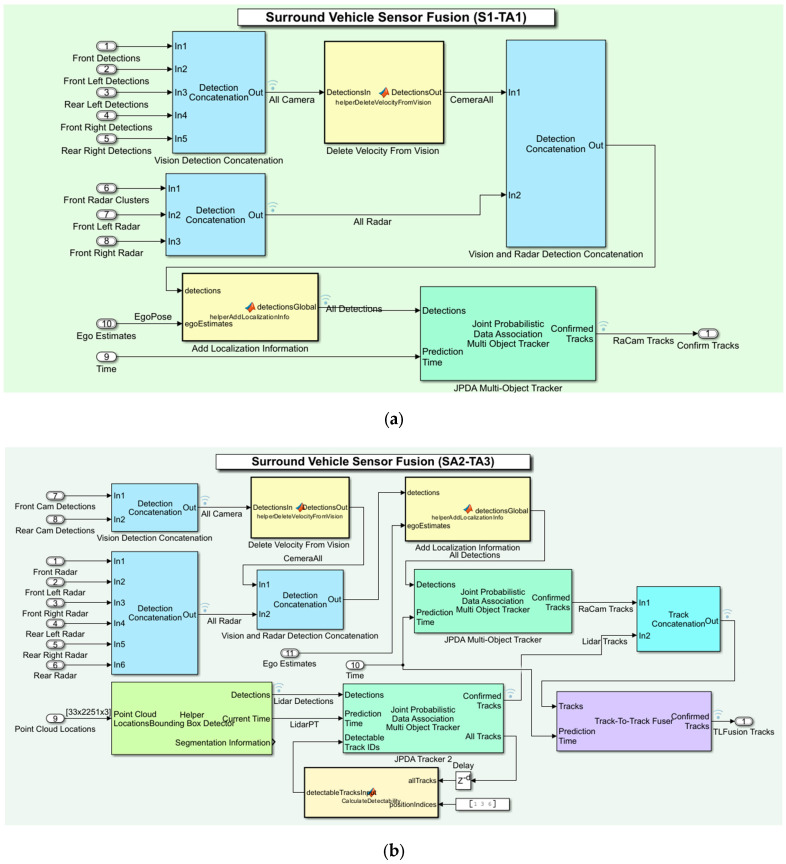
Simulink-based tracking architecture design: (**a**) centralized tracking architecture using radar and camera (S1-TA1); (**b**) decentralized tracking architecture using radar, cameras, and LIDAR (S2-TA3).

The above three architectures and two sensor setups allowed us to experiment with six combinations of sensor fusion and tracking architectures. The two Simulink-based different tracking architectures are presented in Figure 7. For the sake of example, we present two Tas out of six combinations. The centralized TA is shown in Figure 7a, and the track-to-track-level TA is shown in Figure 7b. The joint probabilistic data association (JPDA) tracker in Figure 7a produces the confirmed tracks during the simulation of a trajectory. First, the surround vehicle sensor fusion system receives the sensor data as the input from the driving scenario. Second, this system accumulates each type of sensor data and forwards the signal into a signal bus. Here, five camera detections and three radar sensor data are concatenated using two different detection concatenation blocks provided by Simulink. Before fusing the camera and radar detections using another concatenation block, a customized function is used to return the camera data without velocity information. Third, the fused data are sent to the JPDA tracker by adding the localization information using another customized function. Finally, the JPDA tracker processes this information for each step to produce the confirmed tracks. A similar data flow process with three additional steps can be observed in Figure 7b. The first step involves producing LIDAR detections from LIDAR (point cloud locations) data. The second step produces the confirmed tracks using another JPDA tracker. Third, the two confirmed tracks (LIDAR and radar-camera tracks) are fused using a track concatenation block. Finally, the fused tracks are passed to a track-to-track fuser to produce the central tracks. The following section describes the results of the comprehensive examinations.

In a Simulink model, the detected object’s signal is forwarded to a multi-object tracker to track the target objects surrounded by an ego vehicle. Simulink has a rich collection of multi-sensor-based multi-object trackers [33]. Of the available trackers, the JPDA tracker and the probability hypothesis density (PHD) tracker are the centralized trackers used to track moving vehicles using multiple sensors. The track-to-track fuser supports decentralized sensor fusion. This study performed experiments using a JPDA tracker for centralized tracking and a track-to-track fuser for track-level fusing. The JPDA tracker uses the helperInitializeCVEKFFilter algorithm for radar and camera fusion, whereas LIDAR and camera fusion uses the helperInitLidarCameraFusionFilter algorithm for filtering object detections. The helperInitializeCVEKFFilter is a filtering function that is a modified version of the constant-velocity extended Kalman filter (initcvekf) function provided by MATLAB. This function initializes an extended Kalmal filter tracking object by increasing the velocity covariance for the constant-velocity extended Kalman filter to process the data sensing from fast-moving vehicles. A more detailed description of the extended Kalman filter can be found in Section 3.2.1. Two tracking algorithms, central2sensor and sensor2central, were used for track-level fusion.

## 4. Results

The proposed SMTPE was validated by experimenting with three crash scenarios. The CIREN [17] dataset was used to reconstruct three types of vehicle crashes, namely CIREN-664 (head-on crash), CIREN-816 (rear-end crash), and CIREN-226 (side-impact crash) to evaluate these three sets of tracking performance applying SMTPE. MATLAB, Simulink, and their tools (Driving Scenario Designer app, Navigation toolbox, and Automated Driving Toolbox) were used to reconstruct the crash scenarios. We simulated the crash scenarios using three sensor update rates (200 ms, 100 ms, and 50 ms) to observe the multi-sensor fusion-based data sizes. The data produced from the three sensor update rates are shown in Table 5. The findings from this experiment are as follows:

- The 200 ms sensor update rate produced the smallest data size with the fastest processing for all the planned sensor setups, but it missed a few object detections.
- In contrast, the 50 ms sensor update rate produced the highest data size with detailed detections from the environment of the simulated scenarios.
- Of the three tracking architectures, TA3 (the track-to-track fusion using radar, cameras, and LIDAR) needed the highest processing time compared to TA1 (centralized fusion using radar and cameras) and TA2 (centralized fusion using cameras and LIDAR).

Overall, 100 ms produced the required object detections with optimal data sizes.

**Figure 8:**
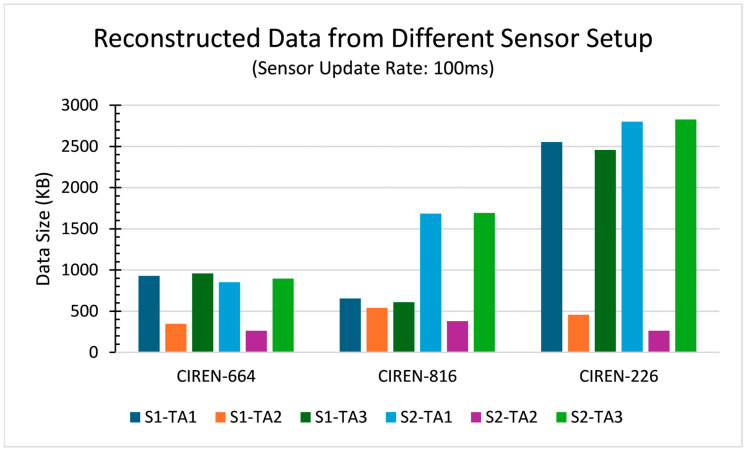
Reconstructed data from multiple sensor arrangements with a 100 ms sensor update rate for CIREN accidents ID 664, 816, and 226.

The data generated from the sensor update rate of 100 ms are compared and presented in Figure 8. For all the scenarios, S2-TA2 (centralized fusion using sensor setup 2, cameras, and LIDAR) produced the lowest data, whereas TA3 (decentralized fusion using radar, cameras, and LIDAR) produced the highest data. Our finding was that the data size from the reconstructed scenarios varied from one to another based on the scenario’s environment and the trajectory time’s duration.

### 4.1. Multi-Sensor-Based Object Detection and Evaluation

**Figure 9:**
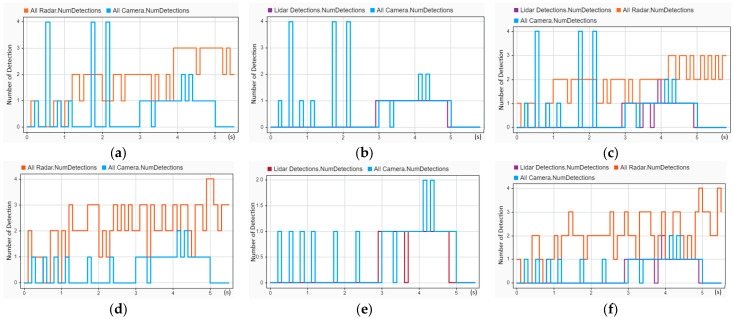
Object detections of the CIREN-664 (head-on crash) scenario using different sensor and tracking architecture setups: (**a**) used S1-TA1; radars have few zero object detections from 0 to 1 s, and cameras have higher zero object detections between 0 and 3 s. (**b**) used S1-TA2; LIDAR has object detections only from 2.9 to 4.9 s. (**c**) used S1-TA3; camera object detections remain the same as (**a**,**b**), whereas radars and LIDAR object detections decrease. (**d**) used S2-TA1; object detection gaps are decreased by radars, and object detection gaps are increased by cameras. (**e**) used S2-TA2; cameras have only a few object detections up to 3 s, and LIDAR can detect objects from 2.9 to 4.8 s. (**f**) used S2-TA3; radar object detections decrease, whereas LIDAR and camera object detections remain the same as (**d**,**e**).

Object detection using sensor data is essential for autopiloting. In present AV technology, a combination of different sensors performs environment perception. Camera sensing data are used to classify the types of objects, such as vehicles, road obstacles, and lane boundaries. Radar object detection is used to generate the kinematic information of the target vehicles. LIDAR delivers high-density three-dimensional (3D) point clouds to reconstruct better 3D detections of objects. Fusing these three types of sensors enhances object detection. Before fusing these three sensors, we first compared the object detections of each sensor in different arrangements using three crash scenarios. The object detection of CIREN-664 (head-on crash) is presented in Figure 9. In Figure 9a–c are the object detections of TA1, TA2, and TA3 using sensor setup 1, whereas Figure 9d–f shows the object detections using sensor setup 2 of three sets of TA1, TA2, and TA3, accordingly. We found that radars have fewer detection gaps from the observed object detection, whereas camera detectors have more. Here, several multiple detections from the same sensor type are observed. For example, as shown in Figure 9a, camera detections reached up to four because sensor setup S1 used five cameras. Similarly, radars have many four-object detections in Figure 9d, where six radars were used for sensor setup S2. Another observation from the LIDAR detections is that there was similar object detection for both sensor setups, S1 and S2, but the detection length was shorter than for the other sensors.

**Figure 10:**
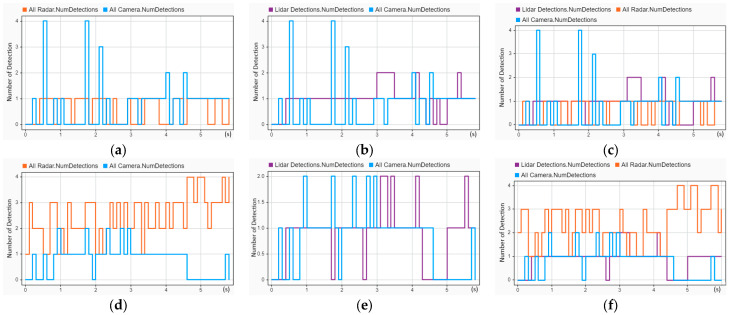
Object detections of the CIREN-816 (read end crash) scenario using different sensor and tracking architecture setups: (**a**) used S1-TA1; front-side radars have many zero object detections from 2 to 4 s, whereas cameras have zero object detections between 1.2 and 2.8 s. (**b**) used S1-TA2; LIDAR has only two zero object detections at 4.6 and 4.8 s, whereas cameras have more zero object detections between 1 and 3 s. (**c**) used S1-TA3; object detections from radars and cameras are almost the same as (**a**,**b**), but LIDAR object detections decrease. (**d**) used S2-TA1; radars have no zero object detections, whereas cameras have zero object detections from 4.5 to 5.8 s. (**e**) used S2-TA2; cameras have zero object detections from 4.5 to 5.8 s, whereas LIDAR is unable to detect objects between 4.3 and 5 s. (**f**) used S2-TA3; radar and camera object detections are almost the same as (**d**,**e**), whereas LIDAR has fewer zero object detections than (**e**).

**Figure 11:**
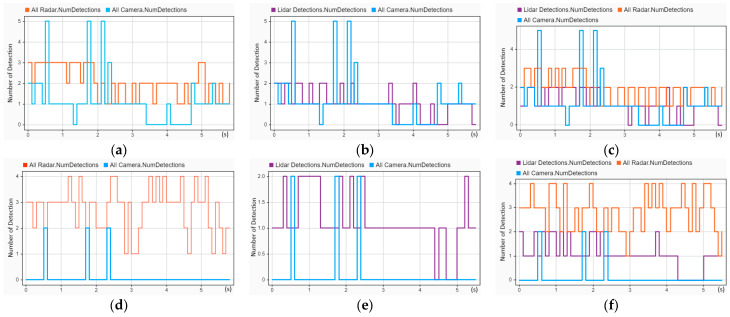
Object detections of the CIREN-226 (side impact crash) scenario using different sensor and tracking architecture setups: (**a**) used S1-TA1; radars have object detections for the complete trajectory duration, but cameras have three-zero object detections starting at 1.3 s, 3.4, and 4.7 s. (**b**) used S1-TA2; cameras have higher object detection gaps than LIDAR from 3.4 to 5 s. (**c**) used S1-TA3; LIDAR, radars, and cameras have almost the same object detections as (**a**,**b**). (**d**) used S2-TA1; radars have no zero detections, whereas cameras have three-time object detections at 0.5, 1.7, and 2.3 s. (**e**) used S2-TA2; LIDAR detects objects for the entire duration of the trajectory but it has zero object detections at 4.4 and 4.7 s. (**f**) used S2-TA3; LIDAR object detections decrease, whereas radar and camera object detections remain the same as (**d**,**e**).

The object detection of CIREN-816 (rear-end crash) is reconstructed in Figure 10. In Figure 10a, radars have frequent gaps in object detections because of the placement of the sensors at the front, front-left, and front-right sides of the ego vehicle. In contrast, in Figure 10d, radars have no zero detection throughout the trajectory. Here, three radars are ranging at the backside of the ego vehicle. Another interesting observation in Figure 11 is that LIDAR detected more in the reconstructed scenario CIREN-226 (side-impact crash) than in the other two crash scenarios.

### 4.2. Evaluation of the Tracking Performance

**Figure 12:**
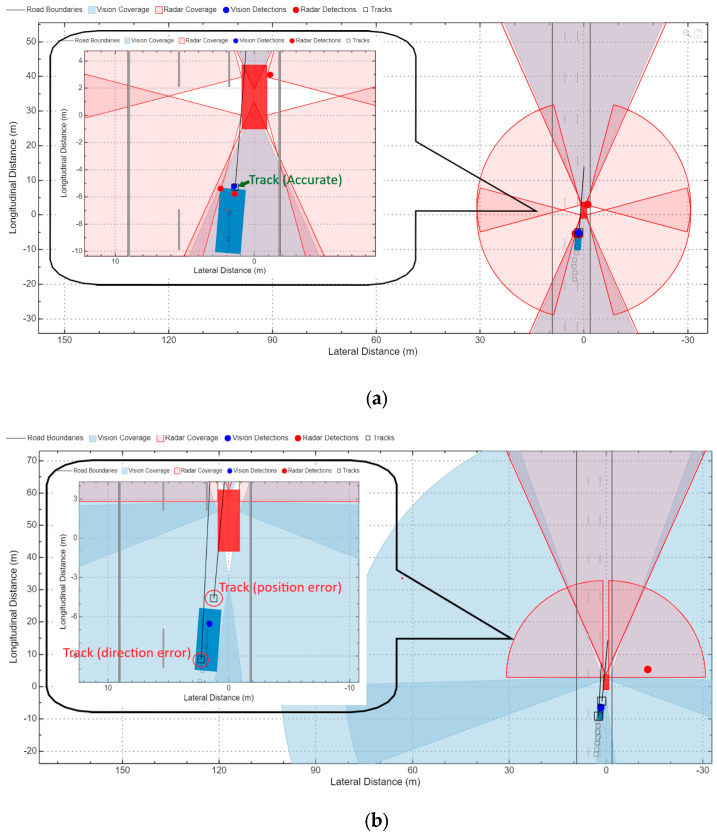
Simulink-based target tracking visualization of the crash scenario CIREN-816: (**a**) accurate target tracked by using the setup S2-TA1; (**b**) error tracks observed by using the setup S1-TA3 and marked red circles with error types.

A visual representation of the target vehicle’s tracking results is presented in Figure 12. The CIREN-816 (rear-end crash) was used to reconstruct this crash scenario. In this recreated crash scenario, the ego vehicle (orange color) used multi-sensor-based surround vehicle sensor fusion to track the target object (blue color). In this case, the tracker performed accurate tracking using the radar and camera fusion setup S2-TA1 (centralized tracking architecture), which is presented in Figure 12a. In contrast, using the exact moment of trajectory but applying sensor setup S1-TA3 (decentralized tracking architecture), the erroneous tracking detections are presented in Figure 12b. Here, the ego vehicle detected two tracks marked as red circles. The track marked in front of the target vehicle has a position error. The other track on the target vehicle has been estimated to be in the wrong direction (the straight line drawn from the track towards the next position of the other vehicle). This visual tracking observation inspired us to perform multiple iterations of in-depth tracking performance evaluation using the proposed SMTPE. The next part of this subsection presents the tracking performance evaluations of the three crash scenarios.

MATLAB provides system objects to expedite tracking performance evaluation for researchers. Such system objects are the OSPA-based trackOSPAMetric object and the GOSPA-based trackGOSPAMetric object. The GOSPA metric includes more features than the OSPA metric, such as localization errors, missed targets, and false track components [34]. These features attract researchers to work with tracking performance evaluation using the GOSPA metric. The GOSPA metric is attracting the attention of researchers, and further improvement initiatives are found in the GOSPA [22,23]. As the GOSPA metric is more beneficial than other tracking performance evaluation metrics, this researcher evaluated the tracking performance for AV crash reconstruction using the GOSPA metric.

**Figure 13:**
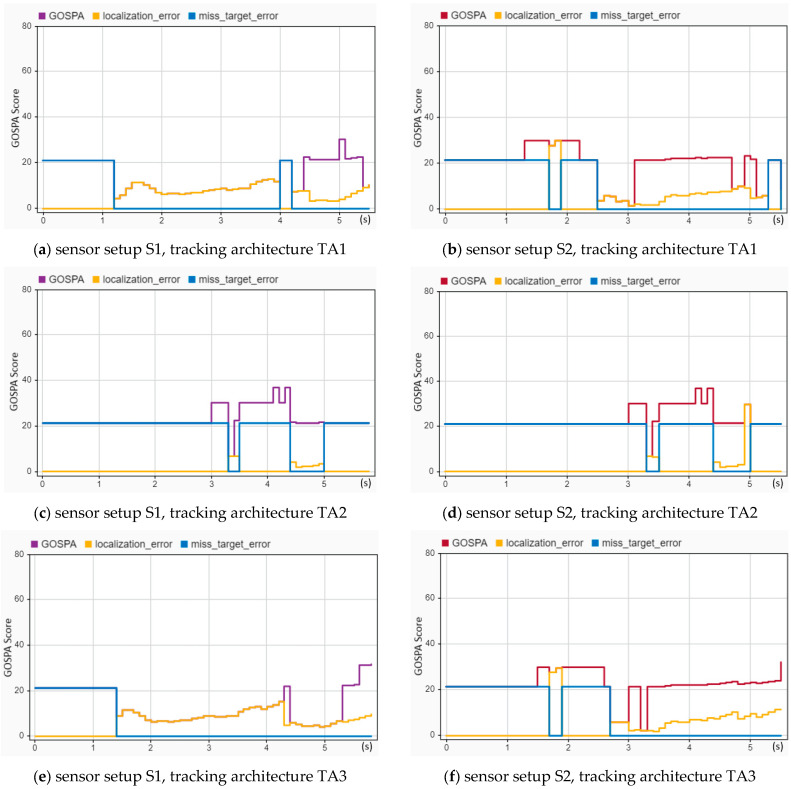
Sensor fusion-based tracking performance assessment of the CIREN-664 (head-on crash) scenario using different sensor and tracking architecture setups: (**a**) used S1-TA1; GOSPA and localization errors are the same from 1.2 to 4.4 s with error values between 8 and 15, but GOSPA errors are more than 20 after 4.4 s; missed target errors are found at 4 to 4.2 s. (**b**) used S2-TA1; localization errors always remain below ten; the GOSPA errors are about 30 from 1.3 to 2.2 s and 23 from 3.2 to 5.2 s; missed target error scores are 21 from 0 to 1.7 s and 1.9 to 2.5 s. (**c**) used S1-TA2; localization errors are zero except for 3.3 and 4.4 s; GOSPA score is above 20, and more than 30 from 3 to 4.4 s. (**d**) used S2-TA2; GOSPA, localization, and missed target errors are almost the same as (**c**). (**e**) used S1-TA3; GOSPA and localization errors are the same from 1.4 to 5.3 s with error values between 5 and 14, and missed target errors are zero from 1.3 s. (**f**) used S2-TA3; GOSPA scores are above 20, whereas localization errors are below 10 after 1 s. See Equation (15) and association equations for formal definitions of GOSPA error, missed targets, and false targets.

The tracking performance of the reconstructed head-on crash (CIREN-664) was evaluated and is presented in Figure 13. In Figure 13a, localization errors (from 1.2 to 4.4 s with error values between 8 and 15) are much higher than the localization errors (below ten for the entire trajectory time) shown in Figure 13b, whereas Figure 13a shows better results for GOSPA (GOSPA scores are more than 20 after 4.4 s) and missed target errors (21 only from 4 to 4.2 s) than Figure 13b (GOSPA scores are 30 from 1.3 to 3.2 s, and 21 from 3.1 to 5.1 s). A similar picture between Figure 13e and Figure 13f can be observed. Additionally, Figure 13b,c are almost identical. Importantly, out of the three tracking architectures, TA1 performed the best for the crash scenario CIREN 664.

**Figure 14:**
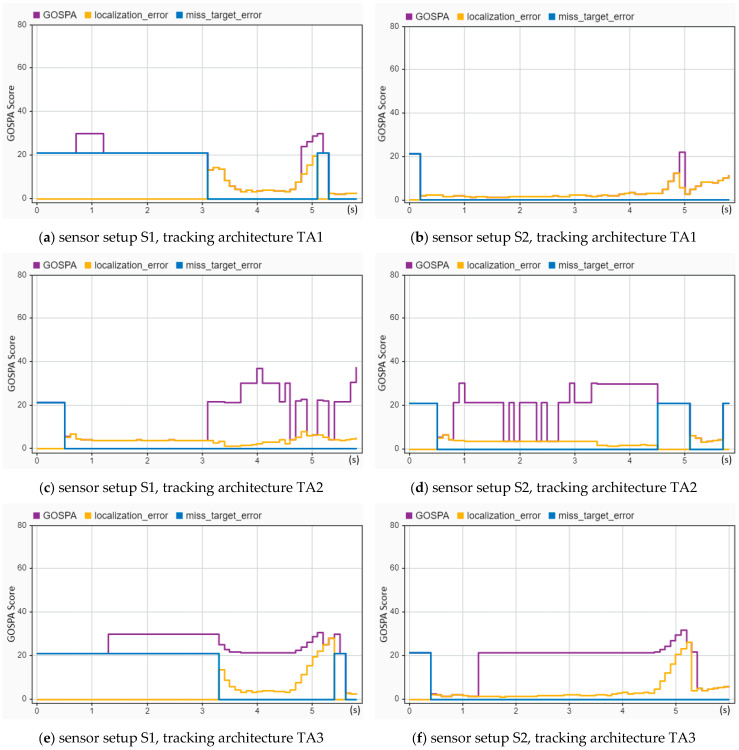
Sensor fusion-based tracking performance assessment of the CIREN-816 (rear-end crash) scenario using different sensor and tracking architecture setups: (**a**) used S1-TA1; missed target errors are 21.5, and localization errors are 0 from 0 to 3.1 s; GOSPA and localization errors are the same from 3.1 to 4.7 s with error values between 5 and 12. (**b**) used S2-TA1; GOSPA and localization errors are the same from 0.2 to 4.9 s with error values below ten, whereas missed target errors remain zero from 0.2 s to the end of the trajectory. (**c**) used S1-TA2; GOSPA and localization errors are the same from 0.5 to 3.1 s and then the GOSPA reaches its highest value of 38 at 4 s. (**d**) used S2-TA2; GOSPA errors are between 20 and 30 from 1.8 to 5.1 s, whereas localization errors are below 8, and missed target errors are zero for the same duration. (**e**) used S1-TA3; GOSPA errors increase to 30 from 20.5 at 1.3 s, remain the same up to 3.3 s, and then fluctuate from 21 to 30 from 3.3 to 5.1 s. (**f**) used S2-TA3; GOSPA errors are 21 from 1.3 to 4.6 s, then rise to 30 at 5.1 s, whereas localization errors remain below 5 from 0.5 to 4.6 s and missed target errors remain zero from 0.5 s.

Using the same tracking architectures (TA1, TA2, and TA3), a rear-end crash (CIREN-816) was reconstructed to evaluate the tracking performance, and the evaluation result is shown in Figure 14. A tracking performance comparison for Figure 14a,b reveals that the GOSPA (30 at 0.7 s and 5 s), localization (reached 20 at 5 s), and missed target error (20.5 in 0 to 3.1 s) scores in Figure 14b are much higher than those in Figure 14a (localization errors are below ten, and missed target errors are zero for the entire time, whereas the GOSPA score is 21 at 4.9 s only). A similar example can be detected for Figure 14e,f. Comparing the TA2 in Figure 14c,d, the GOSPA score and missed target errors reflect better results for Figure 14c. Remarkably, out of the six combinations of tracking architectures and sensor setups, the S2-TA1 performed the best, as illustrated in Figure 14.

**Figure 15:**
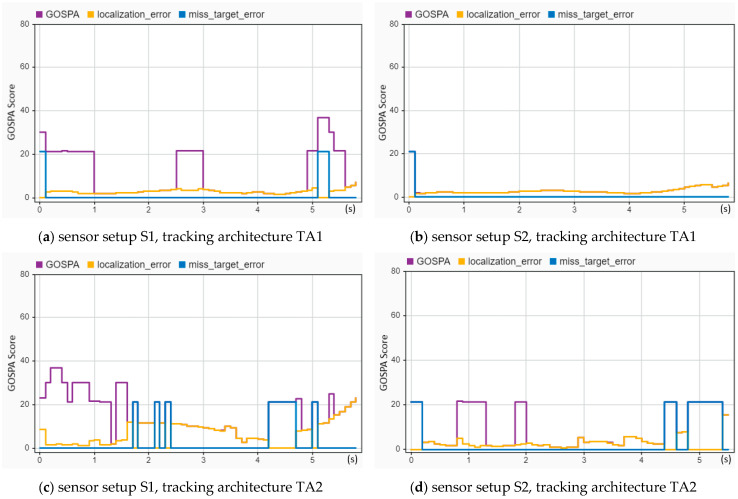

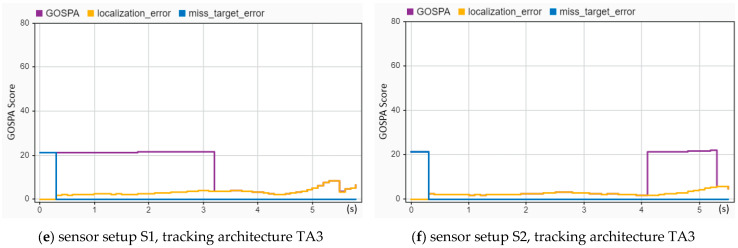
Sensor fusion-based tracking performance assessment of the CIREN-226 (side impact crash) scenario using different sensor and tracking architecture setups: (**a**) used S1-TA1; GOPSA scores are 21 or more from 0 to 1 s, 2.5 to 3 s, and 4.9 to 5.5 s; localization errors are below ten, and missed target errors are zero for the entire trajectory time. (**b**) S2-TA1 has an excellent performance result; missed target errors are zero, and GOSPA and localization errors are below five for the entire trajectory time. (**c**) used S1-TA2; a few instances of GOSPA error scores of more than 30 can be observed from 0 to 1.6 s; localization errors are more than ten from 1.6 to 3.6 s; the missed target error value is 21 from 4.2 to 4.7 s. (**d**) used S2-TA2; multiple instances of an error value of 21 can be observed for GOSPA (0.8–2.4 s, and 1.8 to 2 s) and missed target errors (4.5–4.6 s, 4.8–5.5 s), whereas localization error scores are below ten for the entire trajectory time. (**e**) used S1-TA3; from 0 to 3.2 s, the GOSPA error scores are more than 20, whereas localization errors are less than ten, and missed target errors are zero for all the trajectory time. (**f**) used S2-TA3; GOSPA error scores are more than 20 from 4.1 to 5.3 s, whereas localization and missed target errors are below ten and zero for the entire trajectory time, accordingly.

A much better tracking performance can be observed by comparing all six sensor setups and tracking architectures for reconstructed crash scenario CIREN-226 (side-impact crash) by using the S2-TA1, as illustrated in Figure 15. In Figure 15b, using the S2-TA1, GOSPA scores and localization errors are below five from 0.1 to 5 s (up to the crash moment), whereas missed target errors are zero for the same duration. A few sharp peaks are present in Figure 15a for GOSPA values (20.5 at 0.1–1 s, 21 at 2.5–3 s, and 21 at 4.9 s). In Figure 15c, all these three evaluation variables have higher error values (for instance GOSPA scores are 38 at 0.3 s, 30 at 1.5 s, and 22 at 4.7 s) than in Figure 15d (GOSPA scores are 21 at 0.8 s and 20.5 at 1.9 s). The same performance reflections are observed between Figure 15e,f.

The SMTPE-based evaluation of tracking architecture performance setups is summarized by reporting the highest and the lowest performance of the tracking setups, and their ranks are presented in Table 6. First, we considered two measurement scales and four tracking architecture performance variables to rank the best and the worst tracking performance setups. Then, we used measurement errors, localization errors, false target errors, and GOSPA scores to evaluate the tracking architecture performance. These four variables were then used for grading by applying the error range and their average, considering the total trajectory time of the simulated scenario. This is particularly important because the lower the error value, the better the tracking architecture. Lastly, analyzing the evaluated tracking performance, we found that S2-TA1 was the best tracking architecture, whereas S1-TA2 was the worst. Their GOSPA scores were 2.54 and 15.91, respectively.

## 5. Discussion

In this study, we developed a simulation method (SMTPE) to select a tracking architecture using multi-sensor fusion for AV crash reconstruction. We have shown that SMTPE successfully selected the best tracking setup. S2-TA1, a radar-camera-based centralized architecture of multi-sensor-based surround vehicle sensor fusion, performed the best among the three tracking architectures.

While experimenting with our developed SMTPE, we made many findings that could be worthwhile for further AV developments. Our key findings are presented below.

- The size of the sensor-based data varies based on the driving scenario’s environment and the trajectory time’s duration. The size of the data should be optimal because sensor-based data are supplied for vehicle control and trajectory planning as the input. The SMTPE development process adopted the concept of using the sampling time for better tracking results [14]. We applied three sensor update rates (200 ms, 100 ms, and 50 ms) to decide on the optimal data generated from vehicle sensors. From the analysis of this part of the experiments, 100 ms produced the required object detections with optimal data sizes among these sensors’ update rates. Working with the optimal data generated from the sensors ensures better input for tracking performance enhancement.
- Vehicle tracking performance depends on sensor-based object detection. The proper positioning of the sensors in the vehicle for surround vehicle sensor fusion is essential to achieve better detection coverage. Our comparison between radar, cameras, and LIDAR shows that cameras have a lower object detection than radars for simulated crash scenarios. Our finding concurs with previous knowledge that a sensor’s perception has limitations, which can be mitigated by fusing multiple sensors [8]. Additionally, combining radar, cameras, or LIDAR for sensor fusion can enhance the detection performance for autopiloting [9].
- The SMTPE reveals valuable information that the lower the error value produced from the sensor fusion-based object tracking, the better the tracking architecture. This study has used three tracking architectures to examine all the types of crash scenarios (head-on crash, rear-end crash, and side-impact crash). We evaluated the tracking performance for the AV crash reconstruction using the GOSPA metric [21,22,23] and found that the centralized architecture of multi-sensor-based (radar and cameras) sensor fusion performed the best. The proposed SMTPE is a repeatable simulation method that can be used to decide on the best tracking architecture for future vehicle crash reconstruction and other AV improvement research.

### Guidelines

This research provides guidelines for the best practice in evaluating the tracking performance using multi-sensor-based sensor fusion studies. We emphasize the importance of tracking performance by proposing a standard procedure (SMTPE) for evaluating the tracking performance and selecting the best tracker arrangements for AV developments. This research highlights the following recommendations that raise the scientific rigor when using multi-sensor-based sensor fusion for AV research.

- For the planned experiment, the available datasets or recordings of datasets from a real scenario need to be appropriate.
- The initial setup of a scenario needs to be accurate to produce the required and accurate information from the multi-sensor fusion.
- Precise knowledge is needed to deal with sensing the surroundings in different environments of the trajectories. Indeed, good expertise in the advantages and limitations of sensors is also essential for dealing with real-world environments such as perception at night, rain, fog, or driving on a mountainous road. Similarly, experimenting with different sensors (such as radar, cameras, and LIDAR) with multiple orientation-based setups can produce comprehensive results that can help to select the best experimental setup for planned research.
- Arranging a set of tracking architectures provides comparatively better architecture than experimenting with a single TA. So, it is recommended that tracking performances need to be tested using multiple TA setups.
- Selecting the appropriate tracking algorithm and object tracker requires insight into the tracking algorithm and tracker’s functionalities. Experimenting with different tracking algorithms and trackers can help select a better TA. A tracking metric delivers performance evaluation, and selected tracking assessment metrics must have a higher reputation.
- Determining a suitable tracking threshold value is essential. A performance threshold value (such as setting a 95% confidence interval for tracking performance) can be used to achieve the best tracking architecture. For example, one researcher may set the threshold value of at least 80% accuracy of tracking performance for AV obstacle detection. Similarly, another researcher may wish to experiment with a tracking arrangement that can achieve at least 90% target tracking accuracy for AV collision avoidance. In this research, our predefined threshold values were to accept, at best, 10% errors in a tracking arrangement by setting the factors of GOSPA error, localization error, missed target error, and false detection error at less than or equal to 10%. The tracking performance results support our predefined confidence level for all the factors below 5, as shown in Table 6.
- Finally, it is highly recommended that object tracking performance be evaluated before pursuing any sensor fusion-based AV development to build a higher confidence level.

## 6. Conclusions

Autonomous vehicle (AV) technology developments continue to grow, with the benefits of plentiful resources such as sensors for sensing and tracking algorithms to enhance target following accuracy. This study proposes a novel simulation method for tracking performance evaluation (SMTPE) to solve the issue of how we can select a good tracking architecture for AV crash reconstruction. First, we built three tracking architectures using multi-sensor fusion (a combination of radar, cameras, and LIDAR) to generate tracks from reconstructed crash scenarios using the Crash Injury Research Engineering Network (CIREN) crash dataset. We constructed two centralized tracking architectures and one decentralized tracking architecture. We experimented with two multi-sensor-based surround vehicle sensor fusion setups to test the developed tracking architectures. For surround vehicle sensor fusion, cameras were mainly used to span the surroundings in the first sensor setup, whereas radars performed that task for the second sensor setup. The simulation-based tracks produced by the tracking architectures were then evaluated to determine the best-performing architecture. Our analyzed results show that a centralized tracking architecture fused via radar and cameras using sensor setup 2 constructed the best tracking. This multi-tracking sensor architecture will be used for AV crash reconstruction and forensics. The main contributions of this paper are summarized as follows:

- The most important contribution of this research is that, based on our simulations, a centralized multi-sensor-based surround vehicle fusion tracking architecture (SA2, TA1) is best for reconstructing crashes involving AVs. Our evaluations show that this setup can reconstruct crashes with a high degree of accuracy from data obtained in ideal situations. No extra road infrastructure or external sensor data are required for such reconstruction, thereby adding significantly to the notion of autonomous vehicle crash reconstruction in situations and contexts where such infrastructure is not available.
- We proposed a simulation method to select a good multi-tracking sensor architecture. The proposed method is expected to provide better input for future AV development.
- A further implication of this study is that it will be helpful when reconstructing an AV crash for forensic and investigative purposes, where there is a need for assurance that the sensors have captured all the required information, and that no important information has been lost.
- Finally, in addition to the research findings, brief guidelines are provided for repeating and reusing the proposed method in a similar research domain.

### Challenges and Future Directions

There are a few challenges in the development of multi-tracking sensor architecture. Such challenges are as follows:

- Selecting appropriate sensors and sensor arrangements, such as the number of sensors used from each sensor type, their position on the vehicle, and the required calibration of those sensors.
- The size of the data generated from sensors depends on the tracking architecture and sampling rate used for simulation. Thus, capturing the required information without data loss is challenging.
- Selecting algorithms, filtering functions, object trackers, and tracking architecture requires good knowledge.
- Creating a complete 360-degree surround vehicle sensor fusion is a challenge. To achieve a 360-degree perception, if multiple unnecessary sensors are used, the data produced from the sensors will be larger, hence incurring processing and data storage costs. In contrast, using limited sensors to detect 360-degree surroundings requires a higher angle of view or focal point, which may be impractical.
- Some overlapping of sensor coverage may occur during multi-sensor-based fusion, where inaccurate object detection may occur.

The aim of the paper was to evaluate how different sensor architectures could be useful for reconstructing crashes from simulated data rather than experiment with these different architectures in real crash situations. Such experiments may lead to further changes in sensor architecture and infrastructure dependence due to the problems encountered in real data acquisition and data quality (e.g., noise, corruption, loss). Based on the evaluated tracking performance, the future scope of this study will be using the best multi-tracking sensor architecture for AV crash reconstruction and forensics. A notable feature of SMTPE is repeatable for all other tracking performance evaluations of AV development. It may help for future research in similar sectors, such as unmanned aerial vehicles (UAVs) and autonomous underwater vehicles (AUVs).

## Figures and Tables

**Table 1:** Summary of the related works.

References	Key Contribution	Limitation/Efficacy
[18]	Proposed a metric (C-OSPA) to evaluate multi-target tracking performance	Performed mathematical simulations only
[19]	Developed a tracking performance evaluation method	Used a single sensor for detection
[20]	Algorithms for intelligent trajectory tracking performance evaluation	The perception mechanism was not explained
[14]	Improved path-tracking performance by the proposed algorithm	Absent other vehicles in the experiment scenario
[21,22]	Solved the issues of the OSPA metric and proposed GOSPA metric	Useful for tracking performance evaluation
[23]	Improved the GOSPA metric using temporal dimension specifics	A potential metric for tracking evaluation
[24]	Visual tracking measures for tracking performance	Considered only video data
[25]	Improved the position estimation for vehicle tracking	Only numerical evaluation was performed
[26]	Sensor fusion-based cost-effective vehicle tracking system	Limited to only front-end target tracking

**Table 2:** Sensor setup 1 (S1): the ego vehicle’s sensor arrangements for the surround vehicle sensor fusion.

Sensor	Location	Position (m) [x, y, z]	Rotation (°) [Roll, Pitch, Yaw]	Max. Range (m)
Radar	Front	[3.7, 0, 0.2]	[0, 0, 0]	160
	Front-left	[2.8, 0.9, 0.2]	[0, 0, 45]	30
	Front-right	[2.8, −0.9, 0.2]	[0, 0, −45]	30
Camera	Front	[2.95, 0, 1.1]	[0, 1, 0]	250
	Front-left	[2, 0.9, 0.7]	[0, 1, 65]	80
	Front-right	[2, −0.9, 0.7]	[0, 1, −65]	80
	Rear-left	[2.8, 0.9, 0.7]	[0, 1, 140]	100
	Rear-right	[2.8, −0.9, 0.7]	[0, 1, −140]	100
LIDAR	Center	[1.5, 0, 1.6]	[0 0 0]	120

**Table 3:** Sensor setup 2: the ego vehicle’s sensor arrangements for the surround vehicle sensor fusion.

Sensor	Location	Position (m) [x, y, z]	Rotation (°) [Roll, Pitch, Yaw]	Max. Range (m)
Radar	Front	[1.9, 0, 0.2]	[0, 0, 0]	160
	Front-left	[2.8, 0.9, 0.2]	[0, 0, 60]	30
	Front-right	[2.8, −0.9, 0.2]	[0, 0, −60]	30
	Rear-left	[0, 0.9, 0.2]	[0, 0, 120]	30
	Rear-right	[0, −0.9, 0.2]	[0, 0, −120]	30
	Rear	[0.95, 0, 0.2]	[0, 0, −180]	160
Camera	Front	[2.1, 0, 1.1]	[0, 1, 0]	150
	Rear	[0.56, −0.9, 1.1]	[0, 1, −180]	150
LIDAR	Center	[1.5, 0, 1.6]	[0 0 0]	120

**Table 4:** Tracking architecture (TA) for the sensors’ data fusion.

TA	Fusion Type	Tracker	Filtering Function	Sensors
TA-1	Centralized	JPDA Tracker	helperInitializeCVEKFFilter	Radar, Camera
TA-2	Centralized	JPDA Tracker	helperInitLidarCameraFusionFilter	LIDAR, Camera
TA-3	Decentralized	JPDA, Track-To-Track Fuser	central2sensor, sensor2central	Radar, Camera, Lidar

**Table 5:** Generated sensor-based data from reconstructions of vehicle crashes using the CIREN dataset [17].

Sensor Update Rate (ms)	Sensor Setup	TA	Sensor Data (KB) CIREN-664	Sensor Data (KB) CIREN-816	Sensor Data (KB) CIREN-226
200	S1	TA-1	584.63	326.90	1264.81
		TA-2	179.57	283.05	237.63
		TA-3	567.09	339.10	1228.31
	S2	TA-1	421.79	883.29	1374.46
		TA-2	136.50	196.90	136.31
		TA-3	436.51	875.04	1370.06
100	S1	TA-1	929.62	654.08	2551.38
		TA-2	346.61	540.92	456.40
		TA-3	958.03	610.84	2457.92
	S2	TA-1	852.00	1683.90	2800.71
		TA-2	263.99	380.89	264.44
		TA-3	897.13	1692.71	2827.99
50	S1	TA-1	1861.59	1300.67	4980.17
		TA-2	698.62	1077.75	887.63
		TA-3	1877.60	1212.71	4993.81
	S2	TA-1	1664.29	3422.20	5491.92
		TA-2	533.18	757.50	515.96
		TA-3	1705.84	3389.04	5580.13

**Table 6:** Evaluation summary of the highest and lowest tracking architecture (TA) setups’ performance achieved by multi-sensor fusion. The average values of the performance measurement variables (GOSPA score, localization error, and missed target error) are used to rank the experimented tracking architectures.

Rank	Tracking Architecture	Scenario	Average Localization Errors	Average Missed Target Errors	Average False Target Errors	Average GOSPA Score
Best	S2-TA1 (Centralized TA)	CIREN-226	2.54	0	0	2.54
Worst	S1-TA2 (Centralized TA)	CIREN-226	6.22	10.6	10.24	15.91

## Data Availability

This study was evaluated on the publicly available dataset Crash Injury Research Engineering Network (CIREN) (Current), National Highway Traffic Safety Administration, USA. Available online: https://crashviewer.nhtsa.dot.gov/CIREN/SearchFilter#, accessed on 15 January 2024.
